# Inquiring the inter-relationships amongst grain-filling, grain-yield, and grain-quality of Japonica rice at high latitudes of China

**DOI:** 10.3389/fgene.2022.988256

**Published:** 2022-10-20

**Authors:** Muhammad Shahbaz Farooq, Maqsood Ahmed Khaskheli, Muhammad Uzair, Yinlong Xu, Fahad Masood Wattoo, Obaid ur Rehman, Gyilbag Amatus, Hira Fatima, Sher Aslam Khan, Sajid Fiaz, Muhammad Yousuf, Muhammad Ramzan Khan, Naeem Khan, Kotb A. Attia, Sezai Ercisli, Kirill S. Golokhvast

**Affiliations:** ^1^ Institute of Environment and Sustainable Development in Agriculture, Chinese Academy of Agricultural Sciences, Beijing, China; ^2^ State Key Laboratory of Rice Biology, China National Rice Research Institute, Hangzhou, China; ^3^ National Institute for Genomics and Advanced Biotechnology, Islamabad, Pakistan; ^4^ Department of Plant Breeding and Genetics, PMAS- Arid Agriculture University, Rawalpindi, Pakistan; ^5^ Department of Agronomy, University of Agriculture, Faisalabad, Pakistan; ^6^ Department of Plant Breeding and Genetics, The University of Haripur, Haripur, Pakistan; ^7^ Pakistan Agricultural Research Council, Islamabad, Pakistan; ^8^ Department of Agronomy, Institute of Food and Agricultural Sciences, Florida University, Gainesville, FL, United States; ^9^ Department of Biochemistry, College of Science, King Saud University, Riyadh, Saudi Arabia; ^10^ Department of Horticulture, Faculty of Agriculture, Ataturk University, Erzurum, Turkey; ^11^ Siberian Federal Scientific Center of Agrobiotechnology RAS, Krasnoobsk, Russia

**Keywords:** high latitudes, climate variabilities, inter-relationships, grain-filling rate, rice yield, rice quality

## Abstract

The widespread impacts of projected global and regional climate change on rice yield have been investigated by different indirect approaches utilizing various simulation models. However, direct approaches to assess the impacts of climatic variabilities on rice growth and development may provide more reliable evidence to evaluate the effects of climate change on rice productivity. Climate change has substantially impacted rice production in the mid-high latitudes of China, especially in Northeast China (NEC). Climatic variabilities occurring in NEC since the 1970s have resulted in an obvious warming trend, which made this region one of the three major rice-growing regions in China. However, the projections of future climate change have indicated the likelihood of more abrupt and irregular climatic changes, posing threats to rice sustainability in this region. Hence, understanding the self-adaptability and identifying adjustive measures to climate variability in high latitudes has practical significance for establishing a sustainable rice system to sustain future food security in China. A well-managed field study under randomized complete block design (RCBD) was conducted in 2017 and 2018 at two study sites in Harbin and Qiqihar, located in Heilongjiang province in NEC. Four different cultivars were evaluated: Longdao-18, Longdao-21 (longer growth duration), Longjing-21, and Suijing-18 (shorter growth duration) to assess the inter-relationships among grain-filling parameters, grain yield and yield components, and grain quality attributes. To better compare the adaptability mechanisms between grain-filling and yield components, the filling phase was divided into three sub-phases (start, middle, and late). The current study evaluated the formation and accumulation of the assimilates in superior and inferior grains during grain-filling, mainly in the middle sub-phase, which accounted for 59.60% of the yield. The grain yields for Suijing-18, Longjing-21, Longdao-21, and Longdao-18 were 8.02%, 12.78%, 17.19%, and 20.53% higher in Harbin than those in Qiqihar, respectively in 2017, with a similar trend observed in 2018. At Harbin, a higher number of productive tillers was noticed in Suijing-18, with averages of 17 and 15 in 2017 and 2018, respectively. The grain-filling parameters of yield analysis showed that the filling duration in Harbin was conducive to increased yield but the low dry weight of inferior grains was a main factor limiting the yield in Qiqihar. The average protein content values in Harbin were significantly higher (8.54% and 9.13%) than those in Qiqihar (8.34% and 9.14%) in 2017 and 2018, respectively. The amylose content was significantly higher in Harbin (20.03% and 22.27%) than those in Qiqihar (14.44% and 14.67%) in 2017 and 2018, respectively. The chalkiness percentage was higher in Qiqihar, indicating that Harbin produced good quality rice. This study provides more direct evidence of the relative changes in rice grain yield due to changes in grain-filling associated with relative changes in environmental components. These self-adaptability mechanisms to climatic variability and the inter-relationships between grain-filling and grain yield underscore the urgent to investigate and explore measures to improve Japonica rice sustainability, with better adaptation to increasing climatic variabilities. These findings may also be a reference for other global rice regions at high latitudes in addressing the impacts of climate change on future rice sustainability.

## 1 Introduction

Six continents cultivate rice, except for Antarctica owing to its icy conditions year-round. About 90% of the world’s rice is produced in Asia (Wassmann et al., 2009; Krishnan et al., 2011), with 30% occurring in China as the largest producer worldwide (World Health Organization and Food and Agriculture Organization of the United Nations, 2010; Tang et al., 2014). In China, about 95% of rice cultivation is performed through conventional-flooded systems, which play a significant role in climate change (Tang et al., 2014; Katayama et al., 2015). Overall, the world’s population is growing rapidly; in China, rice production must increase by 20% by 2030 to ensure food security for the population (Farooq et al., 2022a, b), if per capita nutrition consumption is adjusted to the current level (Peng et al., 2008). Previous climate changes showed effects at global (Lobell and Field, 2007; Raza et al., 2019) and regional (Li et al., 2008; Schlenker and Roberts, 2009) scales. Previous reports indicate that rice production will decrease in southern China except for areas where supplementary irrigational sources are available, while yield will increase in northern China, assuming moisture needs are met (Rosenzweig et al., 2014).

The atmospheric temperature has been projected to increase by 1°C–3°C from current conditions by the end of the 21st century (Howe et al., 2019). Three major agents cause climate change: natural factors, human-caused changes (GHGs and CH_4_ emissions), and land-use changes. Human-based activities have resulted in an increased atmospheric concentration of carbon dioxide (CO_2_) from 284 to 410 ppm from 1832 to 2013 (Howe et al., 2019), leading to global warming. Climatic variability analyses for China have shown that overall climate changes became more rapid since the 1950s due to several factors, including abrupt temperature variations toward warming (Piao et al., 2010). In Northeast China (NEC), the highest latitudes globally experienced the most evident temperature warming, making it a major rice-growing region in China since the middle of the last century (Liu et al., 2005; Hu et al., 2019). In NEC, most of the obvious warming has been observed since the 1980s, with annual mean increases of 1.0–2.5°C relative to the previous decade (1960–1970). Decreased annual rainfall has also been observed, especially in summer, since the mid-1960s (Liu et al., 2005), while a decreasing trend in winter temperatures has caused winter warming (Samol et al., 2015). In NEC, the temperature showed a warming trend during the 1920s; then, after three decades, the temperatures started to decrease before increasing again during the 1970s–1980s (Masutomi et al., 2009). Globally and in China, semi-arid regions have become more vulnerable to climatic stresses due to drought stress following continuous decreases in water resources. In NEC, the daily maximum and minimum temperatures have changed significantly, with the former changing more drastically, which had greatly constricted the diurnal temperature values (Asseng et al., 2015).

Temperature variations toward warming or cooling greatly affect the grain-filling phase, leading to changes in the final grain yield. Increased warming intensity, frequency, and duration above the normal ranges (22–32°C) at critical growth stages such as anthesis and grain-filling in rice increases the spikelet sterility (Ali et al., 2022a) and shortens the filling duration for superior and inferior grains, thereby reducing grain production (Krishnan et al., 2011). The range of growing degree days (GDD) for a specific rice cultivar at flowering is nearly identical to this range when cultivated at different temperatures between the optimum and base temperatures. The growth of superior or inferior rice grains increases at higher temperatures, with an accompanying shorter filling duration (Oh-e et al., 2007; Hatfield and Prueger, 2015). A strong negative association has been reported between the ripening period and daily mean temperatures; therefore, temperatures above the optimum range will ultimately reduce the grain-filling, although high temperatures increase the grain-filling rate (Virk et al., 2020). Developmental and cellular processes are affected by high temperatures during anthesis, grain-filling, and ripening, which leads to poor grain quality (Fábián et al., 2019). Rice cultivars experiencing continuous high temperatures during the anthesis and grain-filling phases show poor grain-filling and ultimately low grain weight (Krishnan et al., 2007). Moreover, during the grain-filling stage, longer periods of high temperatures increase the demand for assimilates to avoid white kernels (Kobata and Uemuki, 2004; Shi et al., 2017). Meanwhile, longer periods of drought stress also adversely affect the final grain weight in both superior and inferior grains, consequently reducing the grain yield and quality (Alghabari and Ihsan, 2018).

Variations in ambient temperature during the grain-filling stage affect starch accumulation (Ahmed et al., 2008; Zhang et al., 2021) and the amylose-amylopectin contents of the rice endosperm (Jiang et al., 2003; Zhen-Zhen et al., 2015). Prevailing low-temperature stress during the grain-filling stage will prolong the grain-filling period from 32 days to >50 days (Ahmed et al., 2008). At temperature ranges between 12 and 22°C, the grain weight is comparatively stable and less sensitive to low-temperature stress (Ahmed et al., 2008); however, the grain weight decreases at temperatures below this range. Tillering is generally divided into two sub-processes that are collectively termed as the subsequent growth of axillary buds already produced on each leaf axil. High temperature ranges increase the overall tiller number. At 3–5 weeks after sowing, the relative growth and tillering rates are affected slightly; however, temperatures below the minimum (22°C) greatly affect the tillering rate (Peng et al., 2008).

The product of filling efficiency and sink capacity collectively produce the grain yield in cereals (Kato et al., 2007; Zhang et al., 2021). Wider breeding efforts globally have increased the grain sink capacity and maximized the size of sink organs likely to be harvested to further increase the grain yield primarily by enhancing the spikelet number on each panicle (Kato et al., 2007; Zhang et al., 2021). Consequently, cultivars with extra-heavy or large panicles with more spikelets on each panicle have been produced; for example, the “New Plant Type” produced by the International Rice Research Institute (IRRI) (Peng et al., 2008), “hybrid rice,” “super rice”, and super hybrid rice” (Wang and Peng, 2017). These varieties, however, usually do not reach their grain yield potential due to decreased grain-filling, mainly restricted filling rates and higher frequencies of poorly unfilled grains under varying temperature conditions. The grain weight accumulation and filling rate in rice greatly depend on the position of spikelets on the panicles. Typically, superior spikelets with earlier flowering and generally positioned on the apical primary branches show faster grain-filling, thereby producing large and heavy grains. In contrast, inferior spikelets with late flowering and generally situated at the proximal secondary branches show slow grain-filling or sterile spikelets, ultimately producing non-consumable grains (Fu et al., 2013).

There is a research gap to address the possible adaptation mechanisms for rice production systems against climatic stresses at high latitudes that have already been aggravated in face of climate-induced primary stresses like heat or cold temperature stress; shifts in precipitation; and secondary stresses such as drought, soil salinity, and submergence (Wassmann et al., 2009). Due to obvious warming since the 1960s, NEC has become an important rice-growing region in China. However, abrupt and irregular variations in temperature above the optimum range may adversely affect rice yields at high latitudes due to two major principles. First, temperatures exceeding the maximum range in combination with higher atmospheric relative humidity (RH) will result in the production of more sterile spikelets, leading to poor grain quality. Second, nighttime temperature exceeding the maximum may disturb the pathways of grain assimilate accumulation. Therefore, self-adaptability and response mechanisms at high latitudes in China might result in the development of improved rice germplasm with better resistance against climate-induced stresses. There is a need for the comparative assessment of the inter-relationships among rice grain-filling, grain yield, and grain quality with climatic variations at regional scales and specifically at high latitudes, which is currently the main gap in knowledge. Based on its origin, rice is a semiaquatic phylogenetic plant species that provide unique features of susceptibility and adaptation to climatic variability (Mohanty et al., 2013). Thus, there is a need to characterize the response mechanisms related to grain-filling, yield, and quality under different climate conditions at high latitudes to address the adaptation mechanisms for the sustainability of Japonica rice (Shahbaz Farooq et al., 2022). Remarkable risks and vulnerabilities are associated with the Japonica rice system due to irregular climate variabilities at mid-high latitudes. However, developing methods to address the effects of the response mechanisms of Japonica rice during critical growth stages due to environmental variabilities and implementing adaptation strategies can provide a sustainable rice system to invigorate the future widespread adaptation of Japonica rice at high latitudes (Challinor et al., 2007; Farooq et al., 2021).

Adaptation can be defined as social, ecological, and economic adjustments in response to current or projected climate stresses, or as specific changes in structures, processes, and practices to better exploit opportunities for the sustainability of a production system (Kaufmann et al., 2011). Studies at regional levels have been proposed to investigate response mechanisms and develop adaptation measures (Krishnan et al., 2007). Climate change inclusive of frequent disasters, e.g., floods and droughts, are important factors affecting crop systems, although these changes are further influenced by many secondary factors including market availability, policies, technical development, and natural resources. Therefore, to better understand the response mechanisms to different climatic conditions in Japonica rice and assess the inter-relationships among grain-filling rate and duration, yield, and quality, this study had the following objectives: 1) investigate the self-adaptability of different Japonica rice cultivars and the inter-relationships among the attributes of grain-filling, yield and yield components, and grain quality parameters at highest latitudes of China; and 2) identify and evaluate possible adjustments in agronomic approaches for the sustainability of Japonica rice system against the projected climate change in NEC.

## 2 Materials and methods

### 2.1 Study rationale and site descriptions

**FIGURE 1:**
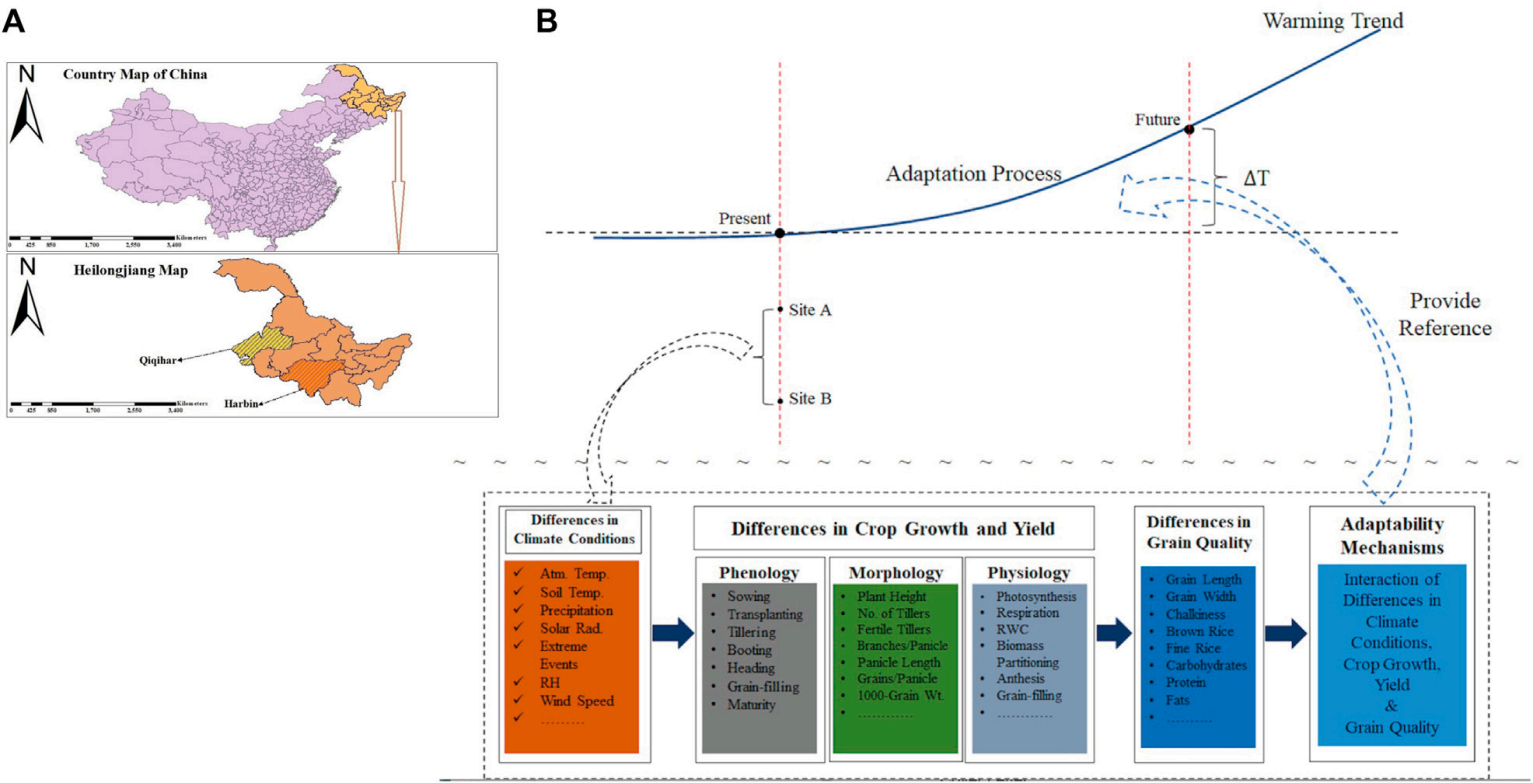
**(A)** Map of the study sites. **(B)** Rationale description of the study (site A: Harbin: site B: Qiqihar; ∆T: change in temperature: Atm Temp: atmospheric temperature; Rad: radiation; RH: relative humidity; RWC: relative water content).

The study rationale was to determine how climatic variabilities are impacting Japonica rice growth, yield, and quality by assessing the inter-relationships among grain-filling, grain yield, and grain quality at high latitudes in China (Figure 1. Vast climate changes, especially climate warming, in the past have favored the expansion of rice cultivation areas in China, especially in Northeast China (NEC). The change in rice production in NEC occurred due to favorable shifts in climatic components such as precipitation and temperature. Climate change also led to natural hazards such as drought, flooding, and increased invasion of insects, pests, diseases, and weeds. NEC has the highest latitude globally and is one of three major rice-growing regions in China. This region is extremely vulnerable to projected climate change regardless of its undistinguished contributions to global warming. China greatly relies on rice cultivation to ensure the country’s food security, employment, and farmer incomes. Rice grain production has shown irregular changes over the last three decades, although the overall production has shown an increasing trend. Enhancing the Japonica rice adaptation process in mid-high latitudes against future projected warming trends compared to the present requires the assessment of the inter-relationships among grain-filling, grain yield, and grain quality in Japonica rice in response to external climatic driving factors. This understanding will inform targeted research and provide evidence for how Japonica rice may adapt at mid-high latitudes in China.

The present study was performed in the Heilongjiang province of NEC, which is situated between 126.6629°E longitude and 45.7421°N latitude. Heilongjiang is the northernmost province of China with a population of 38.17 million and an area of 454,000 km^2^. The climate in this region is a continental monsoon with an annual average temperature ranging between 4°C and –4°C. Winter is generally long and frosty, whereas summer is short and cool. Most of the precipitation is concentrated in summer, with an average annual rainfall of 500–600 mm. Around 59% of the total province area is occupied by mountains. The altitude is low and the interior is relatively flat. Due to obvious climate warming since the last century and measures taken for land reclamation, Heilongjiang has become an important agricultural region for crops such as maize, rice, and sunflower. Two different experimental sites were selected in Heilongjiang province, namely, Harbin and Qiqihar. Harbin, the capital of Heilongjiang province, is situated between 45.7567°N latitude and 126.6424°E longitude. Harbin is dominated by low mountains and hills. The frost-free season lasts 130 days, the annual average rainfall is 400–600 mm, and the mean annual temperature is 3.20°C. Qiqihar, the second largest city in Heilongjiang province, is situated in the west-central part of the province at 47.35°N latitude and 123.91°E longitude. The annual average rainfall is nearly 415 mm, whereas the annual mean surface temperature is 3.95°C. In July, the 24 h average temperature is 23.2°C.

### 2.2 Data source

#### 2.2.1 Experiment design

The present study was performed during the 2017 and 2018 rice-growing seasons. Four different Japonica rice cultivars were selected for the assessment of the impacts of climatic variations on rice grain filling, yield, and quality. The experiments applied a randomized complete block design (RCBD) with three replications. The net plot size was 6 m × 3 m. The cultivars selected for the assessment of impacts of different climates on the grain-filling process and ultimately on grain yield of Japonica rice for this experiment were Longdao-18 (V_1_), Longdao-21 (V_2_), Longjing-21 (V_3_), and Suijing-18 (V_4_). V_1_ and V_2_ are late-maturing cultivars whereas V_3_ and V_4_ are early-maturing. These Japonica rice cultivars were selected because they have been widely cultivated in the Heilongjiang province and have high grain yield capabilities. Cultivars with different growth durations were selected as rice growth duration is critical for determining the optimum plant production. Moreover, the estimation of a change in growth duration and rate during all growth stages due to changes in environmental components is essential for developing a sustainable Japonica rice system under projected climate change at high latitudes. Any additional change in the growth attributes of Japonica rice under varying climatic conditions are well known to drastically change the pliability of crop rotation and intensify the production systems under varying farming arrangements. The local recommended seed rate of 50–60 kg ha^−1^ was used in the rice nurseries for all varieties to attain a balanced planting density. Manual transplantation was conducted by taking 2–3 seedlings per hill at 20 × 20 row spacing and 15 × 15 plant spacing. Manual weeding was carried out three times: 15, 30, and 45 days after transplantation, respectively. A synthetic pesticide (Pendimethalin) was sprayed 8 days after transplantation under optimum field moisture conditions. Macronutrients, i.e., nitrogen (N), phosphorus (P), and potassium (K), were incorporated as basal and splitting doses at local recommended rates of 150–80–80 kg ha^−1^, respectively. Different organic and inorganic N-fertilizer sources were involved viz. synthetic urea (nearly 46% N) and compost manufactured from poultry manure (nearly 1% N). All compost doses were incorporated as basal input during land preparation at 5 t ha^−1^. Synthetic diammonium phosphate (DAP) containing 46% P and 18% N was used for P application. The percentages of N and P provided by compost were also calculated, with the remaining N and P provided using synthetic urea and DAP, respectively. The provision of N from DAP was also calculated and the remaining required N was satisfied using synthetic urea and applied in three balanced splits during land preparation (basal), at tillering, and during panicle initiation. To fulfill the K requirements, muriate of potash (MOP) also known as potassium chloride (KCl) was used, with 60% potash. Synthetic fertilizers for P and K were amended as basal input.

#### 2.2.2 Crop data

For the measurement of yield and yield component data, 1 m^2^ areas were randomly selected from each plot of each block at both study sites to determine the grains per panicle, panicle length, and number of fertile tillers. Meanwhile, 1000 grains were randomly taken from each sub-plot three times and then averaged to estimate the 1000-grain weight for each cultivar. Phenological data records were made for both sites for each growth stage from sowing to harvesting. For the measurement of grain-filling data, 200 panicles were labeled at 0 days in each plot. The sampling for grain-filling data was performed at 1, 5, 10, 15, 20, 25, 30, 35, 40, and 45 days after labeling. Normally, 10 panicles were sampled each time, during which the number of inferior and superior grains was counted at secondary and primary branches, respectively. The rice grains directly positioned on the top three branches (primary) were considered superior grains, while those at the bottom of the three branches (secondary) were termed inferior grains. Later, the superior and inferior grains were oven dried after artificially stripping the hulls for dry weight calculation. The collected data were then fitted using Richard’s growth equation (Richard 1959) with reference to Singh and Jenner (1982) and the growth duration method as described by Zhu and Xianzu (1988).

$$W=A{\left(1+{Be}^{-kt}\right)}^{-1/N},$$ [1]

where W represents the grain weight; A represents the final grain weight; t is the time after anthesis (d); and B, k, and N are equation regression constant parameters. The grain-filling rate of endosperm cells, the average grain-filling rate (GFR_avg_), the maximum grain-filling rate (GFR_max_), the time to reach the maximum grain-filling rate (T_max_), and the grain weight accumulation with the maximum grain-filling rate (W_max_) were calculated as derivatives of the Richard Equation Eq. 1:

$$GFR=AKBer^{-kt}/{\left(1+{Be}^{-kt}\right)}^{\left(N+1\right)/N},$$

$${GFR}_{avg}=\frac{AK}{2\left(N+2\right)},$$

$${GFR}_{\max}=\frac{AK}{{\left(1+N\right)}^{\left(N+1\right)/N}},$$

$$T_{\max}=\frac{\ln B-\ln N}{K},$$

$$W_{\max}={A\left(N+1\right)}^{-1/N},$$

where W is the weight of the grain; A is the final grain weight; t is the time after flowering (d); and B, K, and N are the regression coefficients. The quality parameters were also calculated using standard procedures for brown rice percentage (%), fine rice percentage (%), length–width ratio, chalkiness percentage (%), protein content (%), and amylose content (%).

#### 2.2.3 Meteorological data

The data for environmental variables were recorded during the entire rice crop season by installing automated weather stations at the Harbin and Qiqihar study sites. The weather stations conformed to the specific standards of the China Meteorological Association. Differences in major environmental components were recorded every 5 minutes. The weather data record included the average air temperature (°C), minimum air temperature (°C), maximum air temperature (°C), soil temperature at 5 and 10 cm depths (°C), solar radiation accumulation (MJ/m^2^), relative humidity (%), and daily precipitation (mm).

#### 2.2.4 Statistical analysis

Statistical analysis of the collected data was conducted. Analysis of variance (ANOVA) and Tukey’s HSD test was used at a 0.05 probability level to calculate the relative differences in the means of the cultivars. Considering the difference among treatment means, Duncan’s multiple range test (DMRT) was also used to determine the relative differences between treatment means. The study was conducted under RCBD, where one-way ANOVA was run through Tukey’s HSD test to obtain the differences in the means of the treatments; however, this analysis lacked information on which means differed and to what extent. Therefore, DMRT was applied to identify certain differences between pairs of means. The inter-relationships among grain-filling, yield components, grain quality, and environmental variables were investigated through regression analyses, and the associations were evaluated using partial correlation and correlation analyses using binomial or linear formulas. For the statistical analyses of the collected data, Statistix-8.1, R, and Microsoft Excel 2016 were used. The figures were drawn using SigmaPlot-14.0, Adobe Illustrator, and Microsoft Excel 2016. ArcMap 10.6.1 was used to draw the map of the study sites.

## 3 Results

### 3.1 Rice growth and grain-filling

The cultivars of Japonica rice showed great variations during different growth stages at both study locations. All cultivars showed reduced growth periods in Harbin due to the prevalence of higher temperatures during both years. Suijing-18 was the most affected cultivar, with 89, 61, and 150 and 87, 60, and 147 days from sowing to booting, booting to maturity, and total growing period in 2017 and 2018 growing periods, respectively. The Longjing-21, Longdao-21, and Longdao-18 cultivars also showed shorter growth periods in Harbin compared to those in Qiqihar, with these cultivars showing lower growth periods in Qiqihar by an average of 10 days. The phenological phase variations for all cultivars during both growing periods are shown in Table 1.

**TABLE 1:** Comparability of growing periods (days) of rice cultivars in Harbin and Qiqihar, 2017 and 2018.

Cultivar	Region	Year	Sowing-booting	Booting-maturity	Full growing period
Suijing-18	Harbin	2017	89	61	150
		2018	87	60	147
	Qiqihar	2017	103	57	160
		2018	105	60	165
Longjing-21	Harbin	2017	90	59	149
		2018	87	57	144
	Qiqihar	2017	103	55	158
		2018	106	58	164
Longdao-21	Harbin	2017	100	53	153
		2018	96	51	147
	Qiqihar	2017	107	58	165
		2018	111	61	172
Longdao-18	Harbin	2017	95	57	152
		2018	93	56	149
	Qiqihar	2017	104	58	162
		2018	106	60	166

In the grain-filling process, Richard’s equation showed no clear differences in superior grains in all rice cultivars at both locations. However, in both study seasons, inferior grains showed obvious differences for all cultivars and between rice-growing regions. The weights of superior grains in Suijing-18 were quite similar between Harbin and Qiqihar during the 2017 and 2018 growing seasons (Harbin: 22.91 and 22.76, Qiqihar: 22.70 and 23.01, respectively). Assessment of the variation in weights between seasons showed a modest reduction in grain weight of superior grains in Harbin, while the superior grains of Suijing-18 showed higher weights in Qiqihar. The inferior grains of the same cultivar showed a difference of 5.56 in Qiqihar compared to Harbin. However, superior and inferior grains in Longjing-21 and Longdao-21 did not show significant differences between locations and growth years. However, Harbin showed the best grain weight in all cultivars during both years, while Qiqihar showed increased weights for inferior grains during both study years. Moreover, the superior grains of Longdao-18 had a value of 22.36 in Harbin and 20.88 in Qiqihar in 2017. Overall, the grain weights for all cultivars were slightly increased in Qiqihar compared to Harbin but the best grain-filling weights were observed in Harbin for superior grains. Regarding inferior grains, obvious differences were observed with higher values in Harbin (18.01 and 17.92) compared to those in Qiqihar (14.32 and 14.69) in 2017 and 2018 (Table 2.

**TABLE 2:** Parameter estimation of Richard’s equation in the rice grain-filling process in Harbin and Qiqihar, 2017 and 2018.

Cultivar	Grain type	Region	Year	A	B	K	N	R^2^
Suijing-18	Superior	Harbin	2017	22.91	13.79	0.24	0.55	0.999
			2018	22.70	13.57	0.24	0.55	0.998
		Qiqihar	2017	22.76	0.81	0.60	1.52	0.998
			2018	23.01	0.85	0.63	1.57	0.999
	Inferior	Harbin	2017	20.85	18.77	0.18	0.49	0.999
			2018	20.51	18.91	0.19	0.51	0.998
		Qiqihar	2017	15.28	2.94	0.16	0.28	0.983
			2018	15.51	3.13	0.18	0.31	0.987
Longjing-21	Superior	Harbin	2017	24.03	20.24	0.24	0.66	0.999
			2018	23.87	20.47	0.27	0.69	0.998
		Qiqihar	2017	22.31	1.70	1.28	3.20	0.994
			2018	22.62	1.87	1.37	3.31	0.998
	Inferior	Harbin	2017	21.79	17.35	0.22	0.69	0.999
			2018	21.50	17.14	0.21	0.65	0.994
		Qiqihar	2017	19.59	0.54	0.12	0.08	0.987
			2018	20.01	0.61	0.14	0.10	0.989
Longdao-21	Superior	Harbin	2017	23.780	9.01	0.25	0.60	0.998
			2018	23.57	8.89	0.24	0.59	0.988
		Qiqihar	2017	21.97	1.89	0.21	0.25	0.997
			2018	22.14	1.96	0.25	0.27	0.999
	Inferior	Harbin	2017	19.35	0.74	0.13	0.10	0.996
			2018	19.07	0.66	0.12	0.08	0.993
		Qiqihar	2017	19.53	5.93	0.15	0.38	0.991
			2018	19.87	6.27	0.16	0.38	0.995
Longdao-18	Superior	Harbin	2017	22.36	15.84	0.26	0.69	0.999
			2018	22.11	15.63	0.24	0.63	0.989
		Qiqihar	2017	20.88	0.60	0.14	0.11	0.993
			2018	21.10	0.88	0.17	0.13	0.997
	Inferior	Harbin	2017	18.01	0.80	0.13	0.08	0.997
			2018	17.92	0.79	0.11	0.07	0.995
		Qiqihar	2017	14.32	0.80	0.09	0.09	0.997
			2018	14.69	0.91	0.10	0.10	0.999

A, final grain weight; B, K, N, R^2^, regression coefficients.

We observed that the superior grains of Suijing-18 had higher initial growth at Harbin compared to that in Qiqihar (0.45 vs. 0.40) in 2017. Maximum grain-filling rates of 2.62 mg grain^−1^ day^−1^ and 1.63 mg grain^−1^ day^−1^ were observed in Qiqihar in 2017 and 2018, which were relatively higher than those observed in Harbin (1.63 mg grain^−1^ day^−1^ and 2.88 mg grain^−1^ day^−1^, respectively). In inferior grains comparisons, Qiqihar had lower grain-filling rates (0.78 and 1.68 mg grain^−1^ day^−1^ in 2017 and 2018, respectively) compared to those in Harbin (1.09 and 3.37 mg grain^−1^ day^−1^). However, the superior grains of Suijing-18 in Harbin showed maximum grain weights of 10.31 and 22.80 mg in 2017 and 2018, respectively, compared to 8.44 and 18.70 mg in Qiqihar. In contrast, Qiqihar had a higher average grain-filling rate compared to that in Harbin (1.95 vs. 1.10 mg grain^−1^ day^−1^) in 2017; however, the trend in 2018 differed as the cultivars in Qiqihar showed reduced grain-filling rates while those in Harbin increased incrementally. Meanwhile, superior grains of Suijing-18 required fewer grain-filling days than the inferior grains. Longjing-21 showed initial growths of inferior grain of 1.70 and 0.32 in Qiqihar and Harbin, respectively. Additionally, the superior grains of Longjing-21 showed a higher average grain-filling rate of 2.76 in Qiqihar in 2017, which decreased to 0.88 in 2018, whereas the trend for week grains in Qiqihar was entirely different, as shown in Table 3. The average grain-filling value was higher in comparison to all cultivars and among both locations. In 2017, Longdao-21 showed lower maximum grain-filling rates in Harbin and Qiqihar, at 0.87 and 0.98 mg grain^−1^ day^−1^, respectively (Table 3). Inferior grains in Longdao-18 showed the lowest rate of maximum grain-filling potential of 0.88 and 0.49 mg grain^−1^ day^−1^ in Harbin and Qiqihar, respectively. The inferior grains of this cultivar required the maximum grain-filling periods of 52.60 and 71.49 days among the other cultivars in 2017 and 2018, respectively. The variations in overall grain-filling characteristics in Harbin and Qiqihar in 2017 and 2018 are shown in Table 3.

**TABLE 3:** Comparisons of the rice grain-filling characteristics of Japonica rice under varying climatic conditions in Harbin and Qiqihar, 2017 and 2018.

Cultivar	Grain	Region	Year	GFR_max_ (mg grain^−1^ day^−1^)	T_max_ (days)	Gw (mg)	GFR_avg_ (mg grain^−1^ day^−1^)	GFD (days)	R^2^
Suijing-18	Superior	Harbin	2017	1.63	13.08	10.31	1.11	31.79	0.999
			2018	2.88	12.07	22.80	1.60	30.42	0.999
		Qiqihar	2017	2.62	12.62	08.44	1.96	21.81	0.998
			2018	1.63	17.58	18.70	0.90	20.18	0.989
	Inferior	Harbin	2017	1.09	20.85	09.25	0.74	47.12	0.999
			2018	3.37	10.73	25.38	2.20	46.13	0.995
		Qiqihar	2017	0.78	14.14	05.76	0.53	44.42	0.983
			2018	1.68	16.51	18.41	0.91	43.14	0.993
Longjing-21	Superior	Harbin	2017	1.60	14.48	11.14	1.06	33.94	0.999
			2018	3.31	15.29	25.43	2.35	34.39	0.989
		Qiqihar	2017	3.22	12.58	08.37	2.76	18.15	0.994
			2018	1.67	20.16	22.86	0.88	17.41	0.998
	Inferior	Harbin	2017	1.34	20.18	10.20	0.91	41.13	0.999
			2018	3.52	13.09	24.21	2.35	40.17	0.994
		Qiqihar	2017	0.29	16.57	20.77	0.61	54.06	0.987
			2018	1.50	20.84	19.34	0.85	55.23	0.995
Longdao-21	Superior	Harbin	2017	1.76	10.77	10.89	1.20	28.92	0.999
			2018	1.43	11.90	12.59	1.07	30.18	0.987
		Qiqihar	2017	1.52	09.30	08.39	1.06	32.26	0.997
			2018	1.24	22.05	9.52	0.63	31.11	0.985
	Inferior	Harbin	2017	0.87	16.52	07.35	0.60	52.83	0.995
			2018	2.10	9.24	13.86	1.81	51.42	0.981
		Qiqihar	2017	0.98	17.90	07.61	0.70	50.47	0.992
			2018	1.45	19.73	10.85	0.92	49.17	0.989
Longdao-18	Superior	Harbin	2017	1.64	12.09	10.44	1.11	29.70	0.999
			2018	3.10	12.57	13.48	1.99	30.18	0.993
		Qiqihar	2017	1.08	11.94	07.82	0.75	44.45	0.993
			2018	1.90	16.54	11.35	1.09	43.10	0.977
	Inferior	Harbin	2017	0.88	17.63	06.99	0.61	52.60	0.997
			2018	3.39	12.63	13.76	2.05	51.86	0.997
		Qiqihar	2017	0.49	22.07	05.39	0.34	71.49	0.997
			2018	2.19	14.13	12.75	1.01	70.77	0.987

GFR_max_, maximum grain-filling rate; T_max_, time required to reach the maximum grain-filling rate; GW, final grain weight; GFR_avg_, average grain-filling rate: GFD, active grain-filling duration: R^2^, grain-filling coefficient.

#### 3.1.1 Grain weight accumulation and grain-filling rate

**FIGURE 2:**
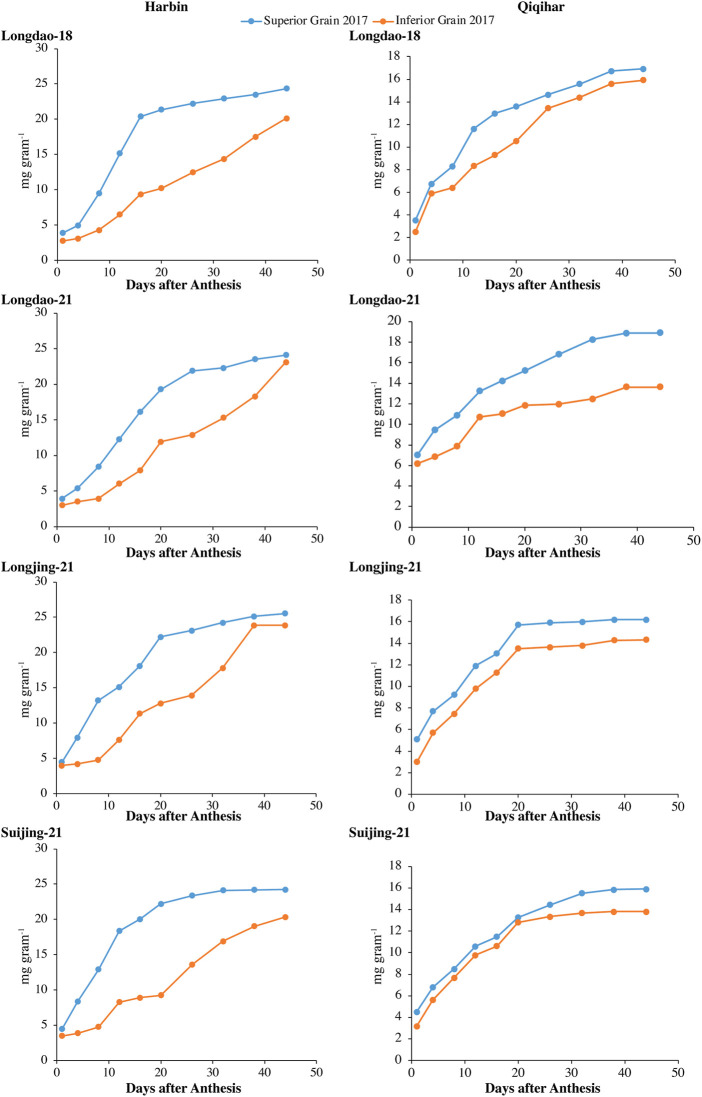
Variations in dry weights (mg grain^−1^) of superior and inferior grains in 2017 in Harbin and Qiqihar.

**FIGURE 3:**
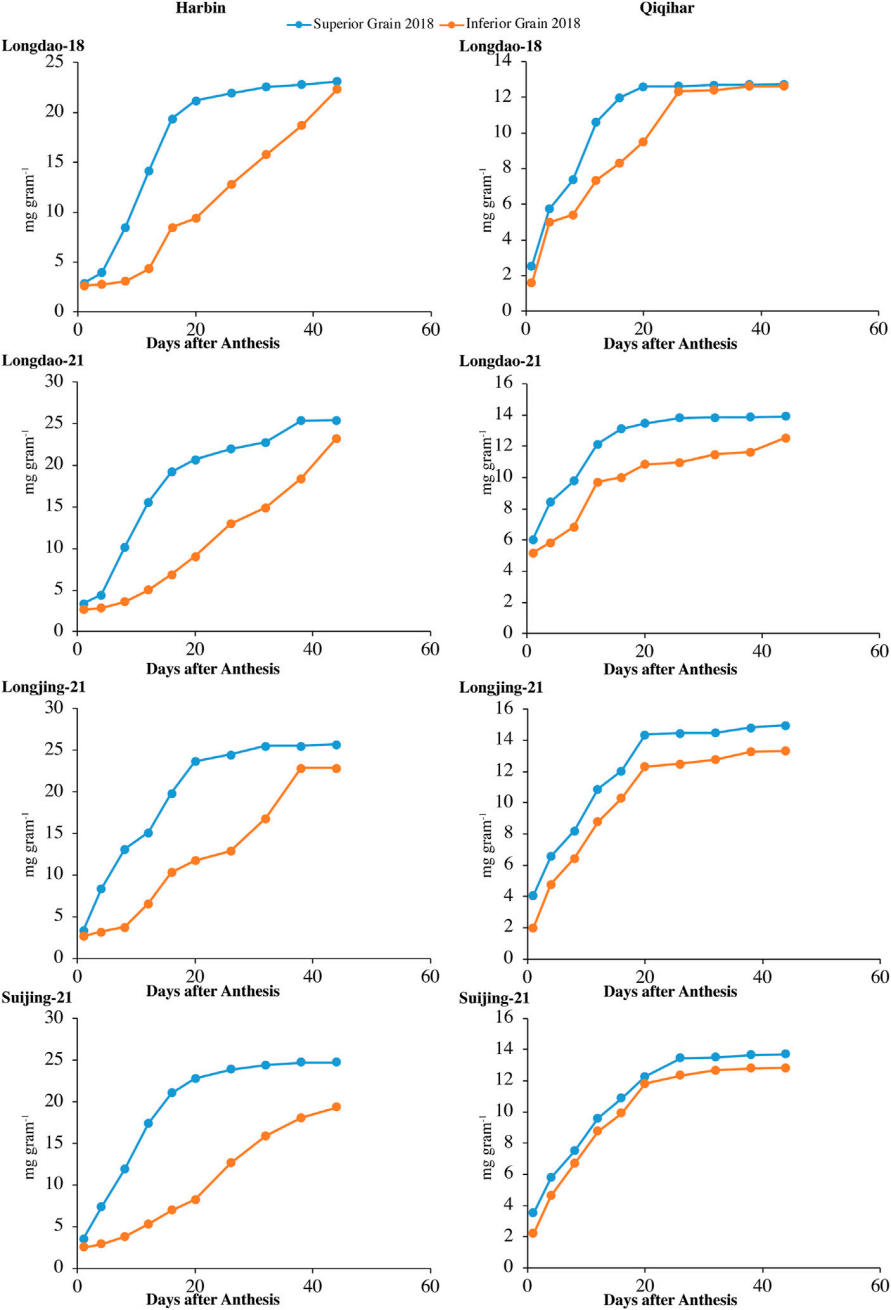
Variations in dry weights (mg grain^−1^) of superior and inferior grains in 2018 in Harbin and Qiqihar.

**FIGURE 4:**
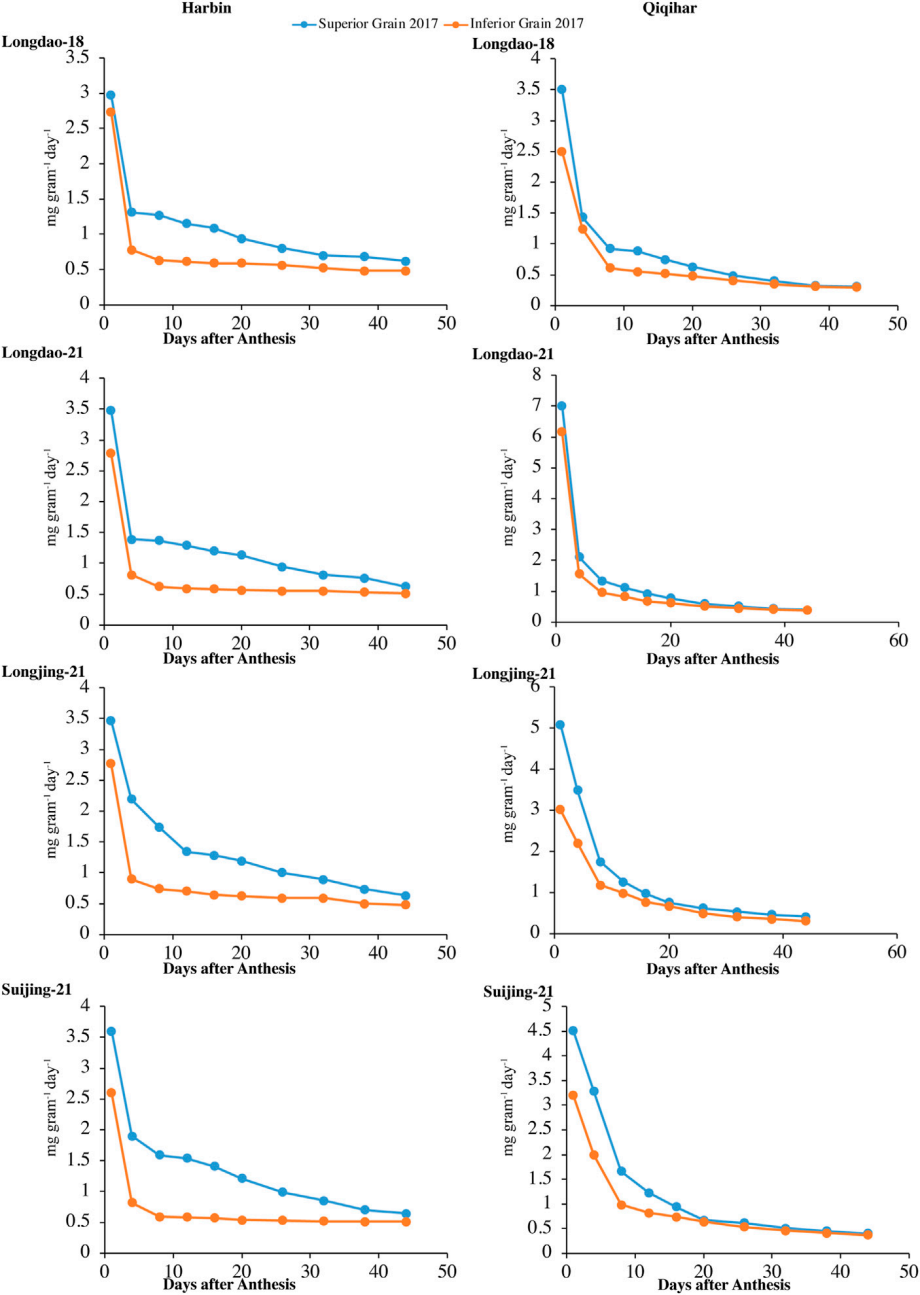
Variations in grain-filling rates (mg grain^−1^ day^−1^) of superior and inferior grains in 2017 in Harbin and Qiqihar.

**FIGURE 5:**
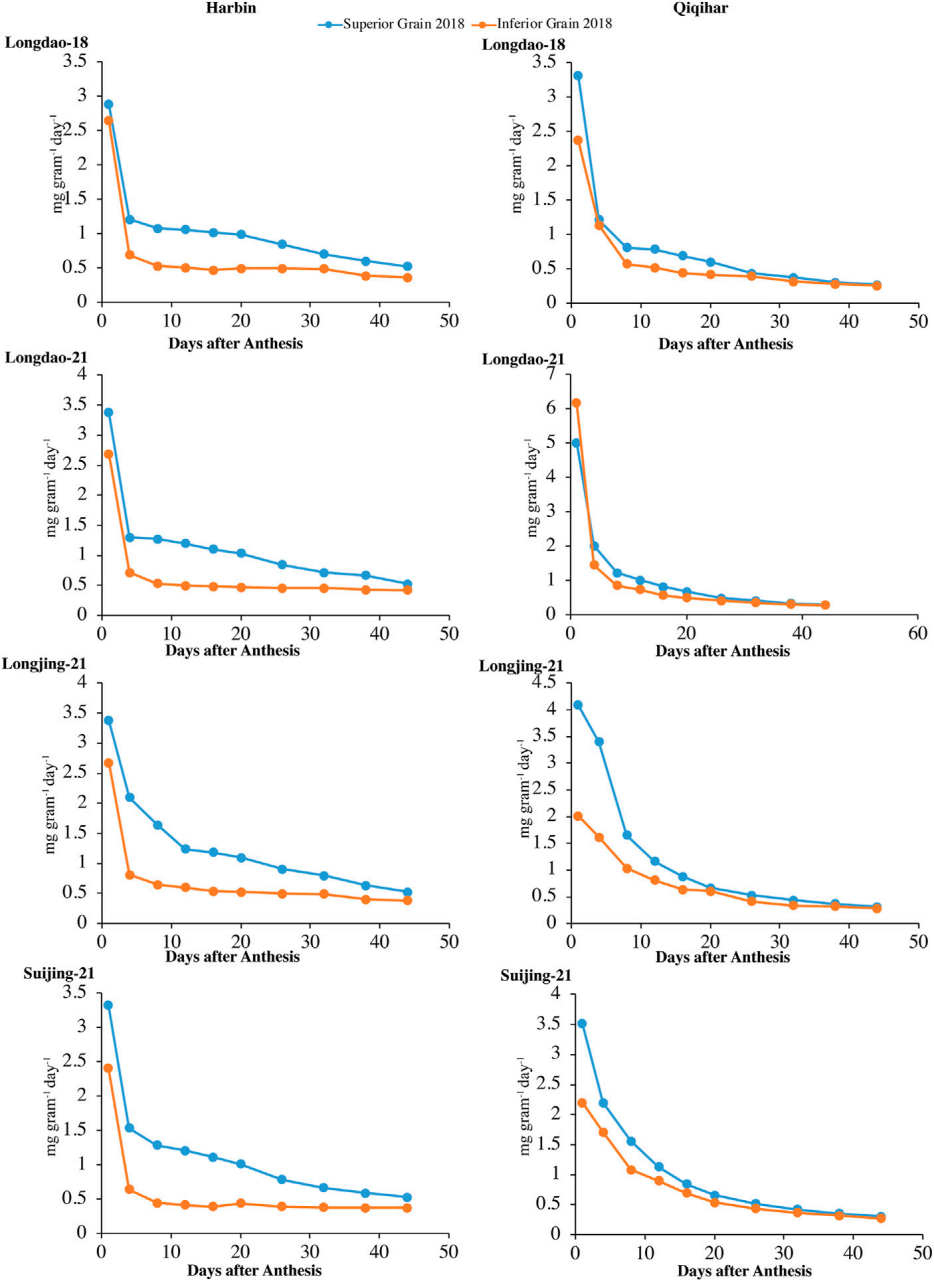
Variation in grain-filling rates (mg grain^−1^ day^−1^) of superior and inferior grains in 2018 in Harbin and Qiqihar.

The dry weight accumulations of superior and inferior grains at both study sites in 2017 and 2018 are shown in Figures 2, 3, respectively. The grain-filling rates at both study sites in 2017 and 2018 are shown in Figures 4, 5, respectively. The dry weight accumulation in superior grains showed an S-shaped trend curve with higher grain-filling rates whereas the dry weight accumulation in inferior grains showed low filling rates. In Harbin, the grain-filling phase overall required 45 days; in contrast, because of the prevalence of stressful environmental conditions during the grain-filling phase, the phase showed a fragmented curve in Qiqihar, although the duration was nearly the same as that in Harbin. During both study years, V_2_ showed a higher trend for dry weight accumulation in both superior and inferior grains, followed by V_3_ and V_4_. Meanwhile, V_1_ showed a reduced dry weight accumulation (Figure 2, 3). However, inferior grains showed an increasing trend during the last weeks of grain-filling for dry weight accumulation, with increased rates during the grain-filling phase and weights nearly the same as those for superior grains at the end of the grain-filling phase in both study regions. Analysis of the weight accumulation among all cultivars in Harbin and Qiqihar in 2018 showed low weight accumulation of the cultivars, with calibrated values of nearly 14 g grain^−1^ for the superior grains.

In Qiqihar, inferior grains had low dry weight accumulation due to unsuitable alterations in the 24-hour mean temperatures during the grain-filling phase, whereas dry weight accumulation was comparatively higher in Harbin in both study years. Moreover, the variation curve showed the same increasing and decreasing trends in both 2017 and 2018; however, the mean dry weights among all cultivars were relatively higher in 2017. The variation extent in environmental parameters prevailed during the grain-filling phase in both study years (Table 4). The dry weights for V_3_ and V_4_ showed different irregular trend curves that were rather S-shaped, with relatively lower grain weight accumulation and grain-filling values during both study years. Inferior grains had low filling rates which could not necessarily be ascribed to varying temperatures experienced by superior and inferior grains, as the maximum and minimum temperatures during different study seasons were relatively constant, generally during 2018. In addition, poor filling rates resulted in slower assimilates accumulation in grain, leading to incomplete and poor grain-filling in inferior grains, causing a constant increase in grain weights until harvest, especially in Qiqihar. Thus, boosting grain yield requires increasing the dry weight accumulation through evaluation of adaptability mechanisms amongst anthesis, grain-filling, and yield at high latitudes to ensure increased filling rates through agronomic and breeding approaches.

**TABLE 4:** Comparison of variations in the prevalence of environmental conditions at the grain-filling stage of Japonica rice in Harbin and Qiqihar, 2017 and 2018.

Genotype	Region	Year	T_avg_ (°C)	T_max_ (°C)	T_min_ (°C)	Daily avg. sunshine (h)	Radiation (MJ/m^2^)	Avg. RH (%)	Avg. soil temp. (5 cm) (°C)	Avg. soil temp. (10 cm) (°C)
Suijing-18	Harbin	2017	20.26	26.01	15.00	6.24	17.64	82.81	21.73	20.32
		2018	19.21	25.34	14.32	6.54	17.93	80.13	22.24	20.97
	Qiqihar	2017	18.44	24.38	12.96	7.30	16.72	80.99	19.77	18.23
		2018	19.67	25.14	13.45	7.41	16.98	78.16	21.05	20.53
Longjing-21	Harbin	2017	20.41	26.10	15.19	6.25	17.71	82.73	21.87	20.45
		2018	21.77	27.18	16.23	6.11	17.93	80.43	22.78	21.04
	Qiqihar	2017	18.62	24.44	13.26	7.16	16.66	81.44	19.91	18.37
		2018	18.93	25.67	14.54	6.98	16.34	79.12	20.45	19.13
Longdao-21	Harbin	2017	19.97	25.68	14.80	5.75	16.81	83.36	21.24	19.84
		2018	20.90	26.81	15.61	6.61	16.09	81.90	21.78	20.12
	Qiqihar	2017	16.91	22.95	11.15	7.74	16.81	77.38	18.66	17.11
		2018	17.54	23.76	13.01	7.11	17.11	79.14	20.07	18.96
Longdao-18	Harbin	2017	20.15	25.92	14.99	5.88	17.09	83.32	21.48	20.06
		2018	21.43	26.12	16.73	6.13	17.56	81.45	22.11	19.76
	Qiqihar	2017	17.71	23.69	12.05	7.31	16.58	79.77	19.27	17.72
		2018	18.32	24.53	14.08	7.79	17.18	80.67	21.03	19.07

Superior and inferior grains showed increased grain-filling rates in all selected cultivars in Harbin until the end of the grain-filling phase and typically showed loop-shaped trend curves during both study years (Figure 4, 5). However, the mean grain-filling rates for all cultivars were lower in 2018 compared to those in 2017. The interaction and reciprocation of grain-filling rates between superior and inferior grains were highly significant in 2018, where the filling rate for superior grains was approximately 2.4 times higher than that in inferior grains. During anthesis, the grain-filling rate in inferior grains was nearly significantly unvarying, as in superior grains. Therefore, almost 24 days from anthesis, the grain-filling rate of inferior grains was comparable to that of superior grains. Temperature variation is a main factor among environmental variables affecting dry weights and filling rates. In Harbin, we observed a significant difference in the variation curves of the dry weights and filling rates between superior and inferior grains; however, both values were constant between grain types 23 days after flowering. The relatively higher assimilates accumulation in Harbin for all cultivars was attributed to the beneficial environmental conditions, including temperature variations.

The optimum growing temperature for paddies during the grain-filling stage ranges between 22°C and 32°C. The average temperature in Harbin during the earlier stages of grain-filling was in the optimum range, which was conducive to earlier increases in dry weight accumulation and grain-filling rates. The time to reach maximum grain-filling varied significantly between superior and inferior grains, indicating that the difference in the prevalence of environmental conditions at different study sites had varying effects on the different cultivars. Among the selected cultivars, the time difference for the maximum filling rates of superior grains to reach the peak value for V_1_ and V_3_ were 7 and 4 days, respectively, while the difference in the time to reach the maximum grain-filling for inferior grains of V_1_ was 6 days at both study sites; thus, the cultivars at Harbin showed an earlier maximum grain-filling compared to that in Qiqihar.

Variations in the environmental components at the study sites showed varying influences on the early- and late-maturing cultivars of the different accumulative temperate regions and greatly influenced the dry weight accumulation in V_4_ and V_3_, while the dry weight accumulation and filling rates were less affected in V_2_ and V_1_ in 2017 and 2018. The grain-filling period was generally divided into three major sub-components (start, middle, and late) to better understand the trends in variation. The overall contributions during the start, middle, and late stages of the grain-filling period were 38.17%, 60.05%, and 28.91%, respectively, in 2018. In 2017, the average contributions varied, with marginal differences in values, at 37.01%, 58.93%, and 30.79%, respectively. Hence, most of the assimilate accumulation in both grain types under varying environmental conditions occurred primarily in the middle sub-phase of the grain-filling stage, comprising nearly 59.93% of grain-filling during both study seasons. The variability among the environmental components during the grain-filling stage in both growing seasons for all cultivars in both sites is shown in Table 4.

#### 3.1.2 Variations in yield and yield-contributing traits

In the current study, the yield-contributing traits also showed great fluctuations along the changes in grain-filling attributes among four different cultivars and at both experimental locations during both study years. The maximum panicle length was longer in Harbin compared to that in Qiqihar for all cultivars except Longdao-21. However, all yield-contributing components showed increasing trends in 2018 as compared to 2017 in both locations. In addition, Suijing-18 produced almost the same number of productive tillers in each plant, while Longjing-21 produced 11 and 16 fertile tillers in Harbin and Qiqihar, respectively. Similar patterns of fertile tiller production were observed in Longdao-21 at both locations; however, Longdao-21 had lower numbers of productive tillers. Longdao-18 showed 8 and 10 productive tillers in Harbin and Qiqihar, respectively, in 2017. A similar trend was observed in 2018, but with increased numbers of fertile tillers. Longdao-18 produced the highest number of grains per panicle (140) in Harbin as compared to the other cultivars in both locations in 2017. In contrast, Longjing-21 produced the lowest number of grains per panicle (88) in Qiqihar compared to the other cultivars. The seed setting percentage also fluctuated among cultivars and study regions. The maximum seed setting percentages of 95.00% and 93.25% were observed for Longjing-21 in Harbin and Qiqihar, respectively, in 2017. The trends in seed setting fluctuations were the same in 2018 but with increased values. Regarding 1000-grain weight, Longdao-21 in Qiqihar showed the maximum value of 28.96 g, while Longdao-18 in Harbin showed the lowest weight of 24.00 g in 2017.

Assessment of variations in overall grain yield showed that Longjing-21 and Longdao-21 produced maximum grain yields of 11235.23 and 11148.42 kg ha^−1^ in Qiqihar and Harbin, respectively, in 2017. In 2018, the grain yields at both study sites were considerably higher than those in 2017 for all cultivars. In 2017, Suijing-18 and Longdao-18 showed the lowest yields of 8985.36 and 8310.67 kg ha^−1^, respectively, in Qiqihar. The results of the comparisons of variation in overall yield components between Harbin and Qiqihar in both study years are shown in Table 5. In contrasting environmental comparisons, all cultivars in Harbin produced long panicles with maximum values of 20.07 and 21.17 cm during 2017 and 2018, respectively. However, Qiqihar showed a higher number of productive tillers, with values of 12 and 14 in 2017 and 2018, respectively. The agro-climatological conditions in Harbin resulted in the production of a higher number of grains per panicle in 2017 and 2018 (121 and 124, respectively) compared to those in Qiqihar (99 and 107, respectively). Moreover, seed setting was also higher in Harbin than that in Qiqihar during 2017 and 2018, which led to higher grain yields of 10160.46 and 10773.51 kg ha^−1^, respectively, in Harbin compared to 9528.75 and 9978.12 kg ha^−1^ in Qiqihar (Table 5).

**TABLE 5:** Comparisons of rice grain yield and yield components under both climatic conditions.

Region		Year	Panicle length (cm)	Productive tillers/plant	Grains/panicle	Seed set (%)	1000-grain weight (g)	Grain yield (kg ha^−1^)
Harbin	Avg. value	2017	20.07	11.00	121.00	0.93	25.53	10160.46
		2018	21.17	13.00	124.00	0.95	26.09	10773.51
	CV (%)	2017	11.18	6.80	11.79	2.53	4.32	7.52
		2018	11.37	7.01	12.13	2.75	4.59	7.83
Qiqihar	Avg. value	2017	18.84	12.00	99.00	0.86	26.90	9528.75
		2018	19.16	14.00	107.00	1.11	28.10	9978.12
	CV (%)	2017	17.39	12.89	9.23	7.64	5.78	5.28
		2018	17.62	13.03	9.52	8.12	6.21	5.73

Therefore, seed setting showed strong negative correlations with the maximum and average grain-filling rates of superior grains in Harbin. The grain yield of superior grains in Harbin was also negatively correlated with the maximum prevailing temperature conditions during both growing seasons. In contrast, the seed setting of superior grains was positively correlated with the maximum and average grain-filling rates during both study years in Qiqihar. Although the grain yield of superior grains in Qiqihar was also negatively correlated, the difference was not statistically significant. Moreover, the 1000-grain weight was also non-significantly and negatively correlated with the maximum and average grain-filling rates of superior grains among all cultivars at Qiqihar (Table 6). Regarding inferior grains, maximum temperature also significantly and negatively affected the grain yields in Harbin. However, the seed setting of inferior grains was positively correlated with the maximum temperature and grain-filling rate in Harbin. The grain yield of superior grains in Harbin was also negatively correlated with the maximum prevailing temperature conditions during both growing seasons. Conversely, seed setting in inferior grains was negatively correlated with the maximum and average grain-filling rates in Qiqihar. Table 7 illustrates the key relationships between the characteristic parameters of inferior grains, yield, and yield components in Harbin and Qiqihar.

**TABLE 6:** Relationships between the grain-filling characteristic parameters of superior grains, yield, and yield components in Harbin and Qiqihar, 2017 and 2018.

Region		Year	R_0_	G_max_	T_max_	W_max_	G_avg_	D	t_1_	t_2_
Harbin	Grains/panicle	2017	−0.02	0.15	−0.60	−0.55	0.13	−0.68	−0.55	−0.62
		2018	−0.01	0.18	−0.53	−0.49	0.18	−0.61	−0.47	−0.59
	Seed set	2017	−0.70	−0.97^a^	0.74	−0.03	−0.97^a^	0.51	0.76	0.71
		2018	−0.63	−0.94^a^	0.79	−0.02	−0.94*	0.58	0.83	0.79
	1000-grain wt	2017	0.13	0.39	0.05	0.70	0.39	0.40	−0.01	0.10
		2018	0.17	0.43	0.07	0.77	0.43	0.43	−0.01	0.13
	Grain yield	2017	0.25	0.84	−0.96^a^	−0.03	0.82	−0.67	−0.97^a^	−0.94
		2018	0.32	0.89	−0.95^a^	−0.02	0.87	−0.63	−0.96^a^	−0.91
Qiqihar	Grains/panicle	2017	0.80	−0.79	0.01	−0.77	−0.81	0.82	−0.61	0.83
		2018	0.83	−0.73	0.02	−0.72	−0.73	0.88	−0.53	0.88
	Seed set	2017	0.14	0.21	0.34	−0.38	0.33	−0.07	0.21	0.26
		2018	0.17	0.25	0.39	−0.35	0.41	−0.05	0.27	0.32
	1000-grain wt	2017	0.45	−0.59	−0.94	−0.19	−0.56	0.53	−0.77	−0.34
		2018	0.48	−0.53	−0.89	−0.18	−0.53	0.58	−0.74	−0.31
	Grain yield	2017	−0.24	−0.13	−0.77	0.57	−0.20	−0.04	−0.29	−0.76
		2018	−0.22	−0.11	−0.71	0.63	−0.19	−0.04	−0.28	−0.73

a Significant at the 0.05 level; R_0_: regression coefficient; GFR_max_, maximum grain-filling rate; T_max_, time to the reach maximum grain-filling rate; W_max_, grain weight at the maximum grain-filling rate; GFR_avg_, average grain-filling rate; D, days to the maximum grain-filling rate; t_1_, t_2_, time between grain-filling phases (start, middle, and late).

**TABLE 7:** Relationships between the grain-filling characteristic parameters of inferior grains, yield, and yield components in Harbin and Qiqihar, 2017 and 2018.

Region		Year	R_0_	GFR_max_	T_max_	W_max_	GFR_avg_	D	t_1_	t_2_
Harbin	Grains/panicle	2017	0.90	−0.84	−0.64	−0.90	−0.84	0.72	−0.70	−0.40
		2018	0.93	−0.81	−0.61	−0.84	−0.79	0.77	−0.67	−0.38
	Seed set	2017	−0.22	0.44	0.49	0.35	0.44	−0.61	0.55	0.31
		2018	−0.21	0.48	0.55	0.39	0.46	−0.57	0.61	0.36
	1000-grain wt	2017	−0.47	0.44	0.07	0.46	0.43	−0.25	0.14	−0.13
		2018	−0.44	0.48	0.09	0.50	0.47	−0.23	0.18	−0.12
	Grain yield	2017	0.85	−0.85	−0.97^a^	−0.87	−0.85	0.91	−0.98^a^	−0.79
		2018	0.88	−0.83	−0.95^a^	−0.84	−0.81	0.93	−0.95^a^	−0.72
Qiqihar	Grains/panicle	2017	−0.43	0.21	0.63	0.56	−0.88	0.66	0.60	0.69
		2018	−0.40	0.26	0.67	0.61	−0.85	0.71	0.62	0.72
	Seed set	2017	0.93	−0.94	0.43	−0.82	−0.21	0.54	−0.01	0.39
		2018	0.94	−0.92	0.47	−0.79	−0.18	0.58	−0.02	0.41
	1000-grain wt	2017	−0.32	0.34	0.45	0.44	0.21	0.18	0.76	0.40
		2018	−0.27	0.38	0.51	0.53	0.27	0.21	0.83	0.45
	Grain yield	2017	−0.75	0.87	−0.41	0.66	0.67	−0.65	0.13	−0.42
		2018	−0.67	0.89	−0.37	0.70	0.74	−0.63	0.15	−0.38

a Significant at the 0.05 level; R_0_: regression coefficient; GFR_max_, maximum grain-filling rate; T_max_, time to reach the maximum grain-filling rate; W_max_, grain weight at the maximum grain-filling rate; GFR_avg_, average grain-filling rate; D, days to the maximum grain-filling rate; t_1_, t_2_, time between grain-filling phases (start, middle, and late).

### 3.2 Grain quality

Table 8 shows that percentage of brown rice did not vary significantly between agro-climatic locations; however, clear variations were observed among cultivars. In 2017, Longdao-21 showed the minimum brown rice percentages, at 81.90% and 81.80% in Harbin and Qiqihar, respectively. The same trend was observed in 2018. In contrast, Longdao-18 showed the maximum brown rice percentages in Harbin and Qiqihar. Additionally, comparisons of the percentages of fine rice showed a variation trend of Longdao-18>Suijing-18>Longjing-21, with the minimum values observed in Longdao-21. In contrast, great variation was observed in whole milled rice percentages. Longjing-21 and Longdao-21 produced almost the same whole-milled rice percentages in Qiqihar and Harbin during both study years while Longjing-21 showed the minimum whole-milled rice percentage in Harbin. Overall, the Harbin site produced lower percentages of whole-milled rice in 2017 and 2018 (58.95% and 57.25%, respectively) compared to those in Qiqihar (67.45% and 69.23%, respectively) (Table 8). Longdao-21 had the maximum length-width ratio of rice grains, followed by Longdao-18>Suijing-18>Longjing-21. The length-width ratios of the rice grains did not differ significantly between sites (Table 7). Chalkiness percentage also varied among experimental locations and cultivars. In 2017, Longjing-21 contained 7.58% and 8.21% chalky grains in Harbin and Qiqihar, respectively, with a similar trend but increased values in 2018. Longdao-21 also showed variations of approximately 2% in both locations.

**TABLE 8:** Variations in rice quality attributes among all four cultivars under the climatic conditions in Harbin and Qiqihar, 2017 and 2018.

Region		Year	Brown rice (%)	Fine rice (%)	Whole-milled (%)	L-W ratio	Chalkiness (%)	Protein (%)	Amylose (%)
Harbin	Avg. value	2017	82.78	70.85	58.95	2.24	2.16	8.54	20.03
		2018	84.31	68.94	57.29	2.19	2.07	9.13	22.27
	CV (%)	2017	1.13	2.70	13.53	11.66	9.72	2.47	5.88
		2018	1.01	3.03	11.33	12.39	11.13	3.29	5.09
Qiqihar	Avg. value	2017	83.03	70.85	67.45	2.26	2.73	8.34	14.44
		2018	82.18	73.11	69.23	2.37	2.41	9.14	14.67
	CV (%)	2017	1.31	1.46	14.31	10.51	10.15	8.18	13.79
		2018	1.45	1.63	12.29	11.98	13.28	7.23	11.13

In this study, the overall values were high for positive quality parameters in Harbin during 2017 and 2018 for all cultivars (Table 8). However, Suijing-18 and Longdao-18 showed distinct variations in chalky grain percentages between study sites. Longdao-18 showed a 3.1% variation in chalkiness percentage at Qiqihar compared to that in Harbin. In contrast, Suijing-18 showed 5.06% variations in Harbin compared to that in Qiqihar. No significant differences in protein percentage were observed between growing locations and among cultivars. However, the amylose percentages varied significantly between study sites. All cultivars grown in Harbin contained significantly higher percentages of amylose contents, with 3%–4% variation compared to those in Qiqihar except Longdao-18, which showed 11.95% variations (Table 8).

**FIGURE 6:**
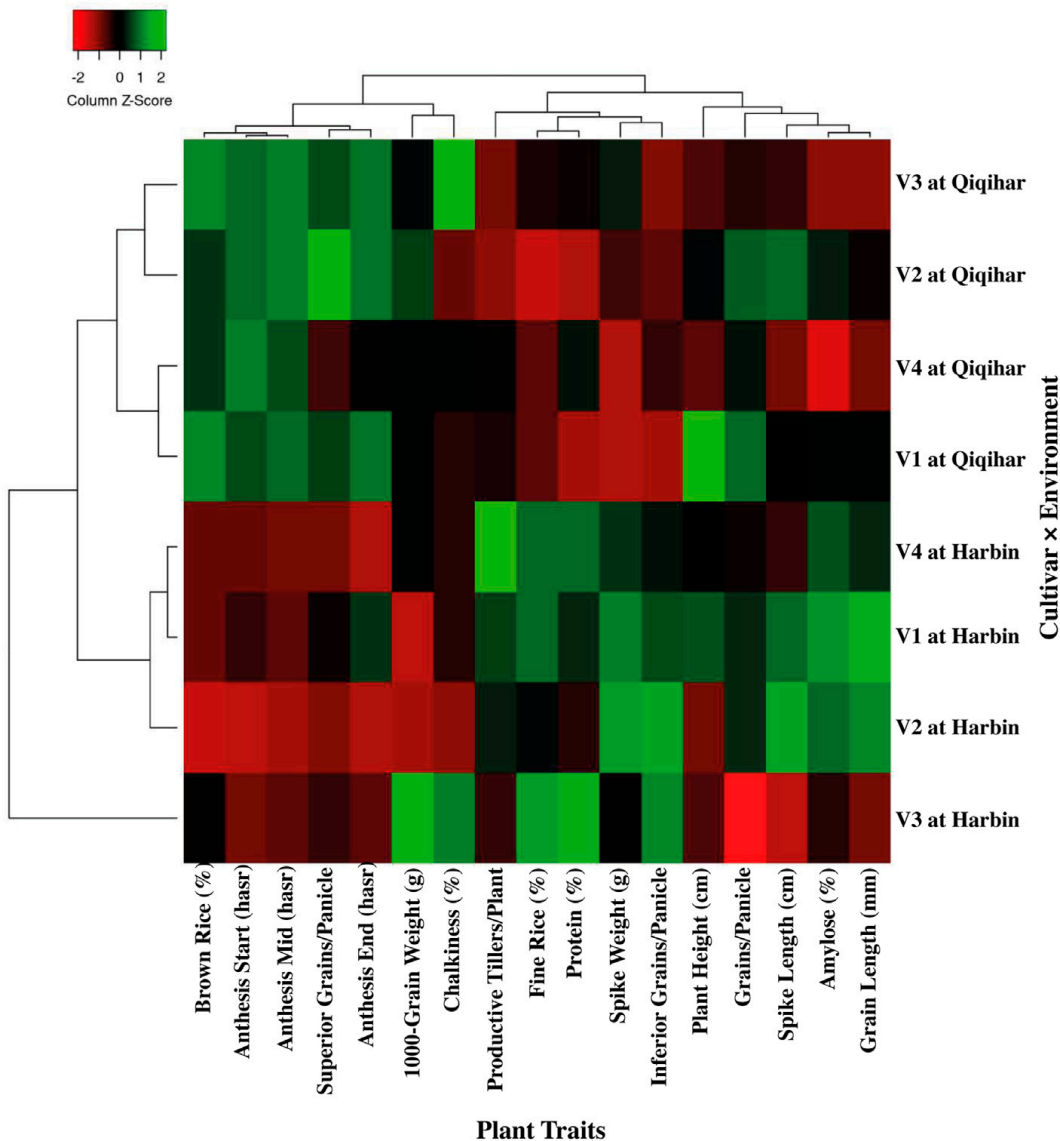
Heatmap describing the two-dimensional visualization of environment-by-trait through clustering of similar and different groups. The dendrogram differentiates the similarities between climatic conditions and plant traits.

Regarding regional variations, chalkiness degree, brown and fine rice percentages, and length-width ratio of rice grains did not show significant changes in all cultivars. However, the percentage of whole-milled rice varied greatly in Qiqihar by 8.50% and 11.50% in 2017 and 2018, respectively, as compared to Harbin. Amylose percentage also varied by 5.86% and 7.50% during 2017 and 2018, respectively, in Harbin compared to Qiqihar. However, protein content and chalkiness percentages varied to a smaller extent at both study sites. Comparison of rice quality between regions showed significantly higher amylose and protein contents in Harbin during both study years and a higher chalkiness percentage in Qiqihar, which indicated a comparatively better rice quality among all cultivars in Harbin compared to that in Qiqihar (Table 8). Based on these data, a clear and more elaborative two-dimensional explanation of the climate-by-trait association through heatmap is shown in Figure 6. This figure is encoded as a pattern of colored boxes to evaluate the relationships between different climatic conditions and rice plant traits. Thus, this heatmap shows the associations between plant traits and climatic conditions to more quickly determine the overall relative changes of traits against environmental conditions to assess the adaptability mechanisms. The heatmap in Figure 6 shows the association between environmental conditions prevailing in Harbin and Qiqihar. The plant traits are shown with dendrograms, with clustering of more similar climatic conditions on one side and grouping of highly associated plant traits on the other side. The columns and rows are ordered to highlight the patterns of traits at Harbin and Qiqihar accompanied by dendrograms.

## 4 Discussion

### 4.1 Rice quality in inferior and superior grains

The grain-filling extent and rate of Japonica rice are mainly determined by the grain position and arrangement in the spikelet on a specific panicle. With higher translocation rates, the weights of superior and large grains on the primary branches are generally increased. In contrast, inferior and light grains positioned on the secondary branches generally have slow translocation rates; therefore, they are considered poorly filled grains and generally undesirable for human use (Xu et al., 2021). Similar observations have been observed in research reporting the significant effects of temperature, maximum grain-filling rate, and average grain-filling rate on grain quality in different climatic conditions (Tables 6, 7). Comparison of both sites in the present study showed a strong negative correlation between maximum temperature and the length-width ratio of superior grains in Harbin. These findings are supported by those reported by Lin et al. (2010), who reported that higher temperature during the grain-filling stage increased the filling rate but deteriorated the quality and reduced the weight of the grains. In Harbin, a strong negative correlation was observed between maximum grain weight and the length-width ratio of superior grains. In contrast, in Qiqihar, the fine rice percentage was positively and significantly more highly associated with the maximum atmospheric temperature. Other quality parameters, including whole-milled rice percentage, chalkiness degree, and length-width ratio, were strongly and negatively associated with the maximum atmospheric temperature. The amylose concentration in superior grains was not correlated with the maximum atmospheric temperature during the entire crop growth but was more obvious during the grain-filling phase in Qiqihar. Moreover, the amylose concentration among superior grains was strongly and positively associated with the maximum grain weight. These findings are consistent with those reported by Ahmed et al. (2008) and Li et al. (2013), who determined that the control of starch in endosperm cells was influenced by environmental and genetic variables during different plant developmental pathways. Increased atmospheric temperature can improve the apparent amylose concentration and could also cause modifications in the primary structure composition of starch granules in endosperm cells such as crystalline, distinct, and grainy shapes, thus resulting in major shifts in the quality and structural composition of storage starches. The dissimilarities in overall amylose concentrations relied primarily on the specificity of rice cultivars; however, changes in the low-temperature conditions broadened the amylose concentration in certain cultivars (Ahmed et al., 2008; Farooq et al., 2021).

In this study, the protein and amylose concentrations in inferior grains in Harbin showed a strong negative association with initial growth. In contrast, the same quality attributes showed strong positive correlations with the maximum temperature in quality assessments of genetic complex control in cereal grains (Sandhu et al., 2021). Similarly, the length-width ratio in inferior grains was also strongly and negatively associated with the prevalence of maximum temperature. Similar results were reported by Zhang et al. (2018), who reported that the amylose concentration in endosperm cells occurred due to the ambient temperature during the early developmental phase, ranging between 5 and 15 days after anthesis at a temperature of 25.8°C. The results of the present study confirmed that maximum temperature was strongly and positively correlated with the chalkiness percentage in inferior grains. The prevalence of heat stress during the grain-filling phase deteriorates the grain quality and undermines the grain yield, with values ranging between 53% and 83% (Ali et al., 2019). Noticeably, the amylose concentration in inferior grains was significantly and positively correlated with the maximum grain weight at Harbin, whereas the protein concentration was significantly correlated with the average grain-filling rate in Qiqihar. Based on these findings, the protein and amylose contents were significantly negatively correlated with the days for inferior grains, as reported by Yang and Zhang (2010). Similarly, Tsukaguchi and Iida (2008) reported that the high temperatures during grain ripening may lead to immoderate morphological features along with pigmentation in grains (Yang et al., 2017), likely due to the reduced activities of enzymes that are essential during grain-filling, the utilization of accumulated assimilates in respiration, and undermined sink activities.

Based on the aforementioned discussion and considering the limited literature on this topic, the grain-filling phase showed strong positive correlations with the environmental conditions in Harbin but negative correlations in Qiqihar due to unsuitable environmental conditions during the grain-filling phase. However, the dry weight values in both inferior and superior grains were highest in V_2_, followed by V_3_ and V_4_, in both study years. The minimum dry weight values were observed in V_1_, as shown in Figures 2, 3. The maximum higher temperature boosted the grain growth rate, which ultimately reduced the grain-filling duration (Oh-e et al., 2007; Hatfield and Prueger, 2015). These findings are consistent with those reported by Tian et al. (2007), who observed that higher maximum temperatures during anthesis and grain-filling reduced the grain yield by inducing higher spikelet sterility and reducing the grain-filling duration. For a specific rice cultivar, the GDD required for anthesis was comparatively the same in different environmental conditions within the range of the base and optimal temperatures (Ali et al., 2022b). These results further confirmed that the dry weights of inferior grains in Qiqihar were significantly lower, as expected, due to shifts in the prevalent temperatures during grain-filling (Chaturvedi et al., 2017; Farooq et al., 2021). Therefore, the dry weights of the grains in Harbin were relatively higher than those in Qiqihar during both study years. The root activities are reduced due to high temperatures, which negatively affect the photosynthetic rate (Shah et al., 2011). The prevalence of higher temperatures during anthesis and grain-filling reduces the grain yield due to increased rates of sterile spikelets and reduced grain-filling duration (Tian et al., 2007; Xie et al., 2009).

The present study evaluated the overall variation in grain yield and yield components between Harbin and Qiqihar in 2017 and 2018, as shown in Table 5. In contrasting environmental comparison, all cultivars showed longer panicles during both study years in the agro-climatic conditions in Harbin compared to those in Qiqihar. However, Qiqihar showed a higher number of productive tillers from each plant. These results are consistent with those reported by Kumar et al. (2021) and Mai et al. (2021), who reported that air temperatures <20°C during the tillering phase were related to an increased number of panicles. Proportionately, the productive tillering capacity of a specific variety determines the overall yield potential; however, cultivars with more tillering capacity may exhibit a large repugnance in the transportation of assimilatory products and other essential nutrients, leading to major changes in grain development and, ultimately, grain yield and quality. The optimum temperature required for grain ripening is less than that for tillering and flowering (Oh-e et al., 2007). The panicle weight decreases under continuous high temperatures. Moreover, a decrease in dry matter accumulation in the panicle was reported after high temperature stress (Kobayashi et al., 2006; Chen et al., 2017), at least partly due to the higher rate of spikelet sterility. Therefore, the dry weights of panicles would not necessarily increase the accumulation of assimilatory products in culms and leaves; however, the environmental conditions were suitable for panicle development. Increased tillering capacity has been reported with rising temperatures (15–33°C), with values outside these ranges unfavorable for tillering (Ghadirnezhad and Fallah, 2014; Fahad et al., 2017). Furthermore, larger numbers of tillers have been observed during the early growth stages under high-temperature conditions within the maximum adaptable ranges. Moreover, the maximum tillering capacity of a potential cultivar was relatively earlier over optimum temperature conditions. Similar results were reported by Oh-e et al. (2007), who suggested that at maturity, the number of tillers was lower under high-temperature conditions compared to that in ambient conditions for crops grown in a temperature gradient chamber.

### 4.2 Relationships between environmental factors and rice growth periods

**FIGURE 7:**
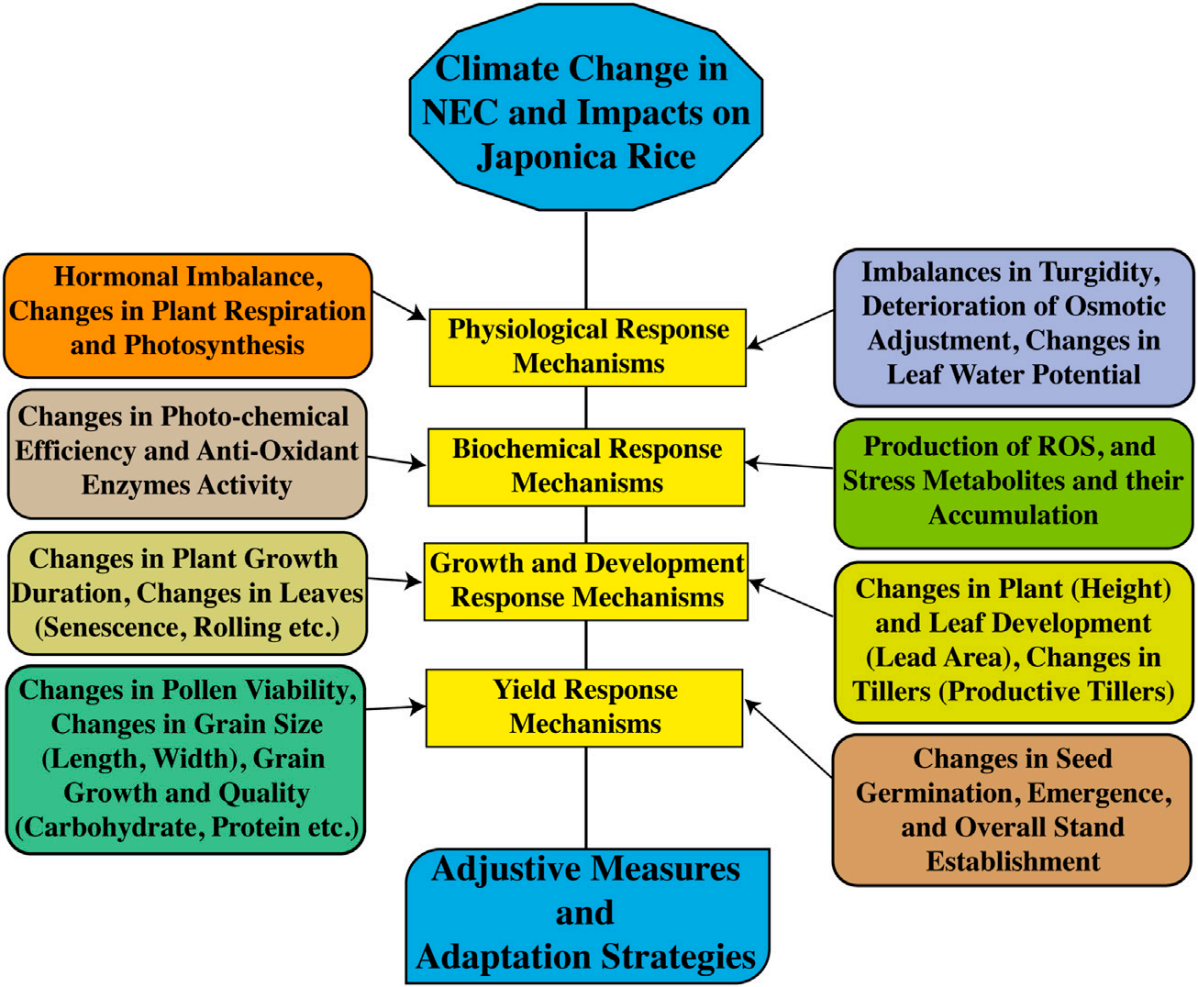
Roadmap for sustainable production of Japonica rice in NEC through an assessment of response mechanisms, thereby disseminating adjustive measures.

One possible future threat to rice quality and production is alternations in climate. Such changes affecting rice growth, physiology, morphology, and phenology might lead to serious food security threats due to decreased rice production worldwide (Krishnan et al., 2011; Farooq et al., 2022b). Assessments of the relationships between environmental factors and the growth periods of rice crops showed higher variations at both experimental locations. The increasing surface temperature affected the growth phases of the rice crop. During the early growth stages, average temperature, solar radiation hours, radiation accumulation, and RH did not show significant effects during the initial growth cycle (from transplanting to booting) of the crops at Harbin and Qiqihar, similar to the results reported by Farooq et al. (2021) and Shahbaz Farooq et al. (2022). However, these environmental variables showed a negative relationship with the early specific growth phase of the plant. Variation in environmental variables significantly affected the later growth phase in both Harbin and Qiqihar. Average sunshine hours, daily radiation average soil temperature (0–5, 5–10 cm) showed strong negative correlations from booting to maturity cycle of the crop in Harbin. However, RH was positively associated with the later growth phase of the plant in Harbin. In contrast, in Qiqihar, daily average sunshine hours showed a significant effect (r = 0.958*) between booting and maturity. Daily average solar radiation also showed an effect, although the effect was not statistically significant. These were consistent with those reported by Yang et al. (2017), who reported that shifts in the average daily day and night temperatures along with remaining environmental variables influenced the overall growth by undermining the physiological processes, grain yield and yield components, and grain quality due to high-temperature stress. The presence of high day or nighttime temperatures during anthesis and grain-filling reduces grain quality (Morita et al., 2004; Wada et al., 2021). Based on the aforementioned discussion and findings of this study, Figure 7 represents the sustainability of Japonica rice in NEC through investigations of response mechanisms in local regions, although this study did not consider all response mechanisms.

## 5 Conclusion

The results of this study showed the changes in rice grain yield due to changes in grain-filling components because of relative changes in environmental components associated with climate change. The phenological, morphological, and physiological response mechanisms that consequently induced the observed changes in grain yield and quality under the differential impacts of changes in environmental components at high latitudes are still unknown. This study identified inter- and intra-varietal variations in different environments due to the changes induced by fluctuations in the environment-driven factors. These changes mainly affected the maximum and average grain-filling rates as well as grain quality in superior and inferior grains. Based on the adaptability mechanisms tested during the 2-year study period, Japonica rice cultivars with shorter growth duration are recommended for mid-high-latitudes of China to avoid the effects of environmental stresses on later developmental stages, especially anthesis and grain-filling. The findings of this study should also be extended to assess the adaptability mechanism of Japonica rice in response to the combination of more than two environmental components to improve the adaptation of these crop plants to climate stresses. Moreover, the results of this study suggested the need to test different Japonica rice cultivars in the same agro-climatic regions to identify heat tolerance mechanisms for future prospects, Japonica rice sustainability, and profitability at high latitudes. The findings of this study underscored the dire need for modern and innovative methods of growing degree days (GDD) to estimate the effects of temperature variations on critical and major growth phases such as anthesis and grain-filling and to observe the interannual shifts as modern GDD measures consider various thresholds of the environmental components.

Our study investigated the responses of commercial Japonica rice cultivars to varying environmental variables under field conditions and observed their capacity and resilience to withstand changes in environmental conditions. Our results on the change in grain yield and quality reinforce the dire need for additional measures in developing rice production systems with both high yield and tolerance to climate stress with less susceptibility and better resilience to climate change. Future research focus should not be limited to only the anthesis and grain-filling phases, as the pre-flowering growth stages such as from panicle initiation to anthesis, etc. also appeared to be susceptible to changes in environmental conditions. Hence, given these adaptability mechanisms to different climatic conditions, the investigation and exploration of additional measures to improve Japonica rice sustainability with better adaptation to increasingly climatic variabilities are urgently required. Moreover, research-based investigations to explore the mechanisms behind spikelet fertility and differentiation under varying environmental conditions are also needed. Additionally, further refinements are needed in the crop-growth models currently available for the deep assessment of the relative differential impacts of changed climatic conditions on crop morphology, physiology, phenology, and yield, which require more accurate simulations related to the differential influences under climate change scenarios. The findings of the current study also emphasize the need for increased awareness of the impacts of the relative contributions of environmental components on physiological and biochemical pathways regulating plant growth and development.

## Data Availability

The original contributions presented in the study are included in the article/supplementary material. Further inquiries can be directed to the corresponding authors.
